# Combination of melt-electrospun poly-ε-caprolactone scaffolds and hepatocyte-like cells from footprint-free hiPSCs to create 3D biohybrid constructs for liver tissue engineering

**DOI:** 10.1038/s41598-023-49117-x

**Published:** 2023-12-13

**Authors:** Josefin Weber, Carsten Linti, Christiane Lörch, Marbod Weber, Madelene Andt, Christian Schlensak, Hans Peter Wendel, Michael Doser, Meltem Avci-Adali

**Affiliations:** 1 Department of Thoracic and Cardiovascular Surgery, University Hospital Tuebingen, Calwerstraße 7/1, 72076 Tuebingen, Germany; 2 Biomedical Engineering, German Institutes of Textile and Fiber Research Denkendorf DITF, Körschtalstraße 26, 73770 Denkendorf, Germany

**Keywords:** Induced pluripotent stem cells, Tissue engineering, Diseases, Extracellular matrix

## Abstract

The liver is a vital organ with numerous functions, including metabolic functions, detoxification, and the synthesis of secretory proteins. The increasing prevalence of liver diseases requires the development of effective treatments, models, and regenerative approaches. The field of liver tissue engineering represents a significant advance in overcoming these challenges. In this study, 3D biohybrid constructs were created by combining hepatocyte-like cells (HLCs) derived from patient-specific footprint-free human induced pluripotent stem cells (hiPSCs) and 3D melt-electrospun poly-ε-caprolactone (PCL) scaffolds. First, a differentiation procedure was established to obtain autologous HCLs from hiPSCs reprogrammed from renal epithelial cells using self-replicating mRNA. The obtained cells expressed hepatocyte-specific markers and exhibited important hepatocyte functions, such as albumin synthesis, cytochrome P450 activity, glycogen storage, and indocyanine green metabolism. Biocompatible PCL scaffolds were fabricated by melt-electrospinning and seeded with pre-differentiated hepatoblasts, which uniformly attached to the fibers of the scaffolds and successfully matured into HLCs. The use of patient-specific, footprint-free hiPSC-derived HLCs represents a promising cell source for personalized liver regeneration strategies. In combination with biocompatible 3D scaffolds, this innovative approach has a broader range of applications spanning liver tissue engineering, drug testing and discovery, and disease modeling.

## Introduction

As the largest solid internal organ of the body, the liver fulfills various functions that are essential for health and longevity, such as the production of plasma proteins, e.g. albumin, fibrinogen, apolipoproteins, coagulation factors, anti-trypsin, plasminogen, transferrin, or retinol-binding protein. In addition, the liver is essential for bile secretion and drug detoxification^1^, it regulates the blood volume, supports the immune system, and controls several metabolic processes through endocrine signaling. Viral infections, autoimmune or hereditary diseases can lead to liver failure. In 2017, approximately 2.14 million deaths were attributed to liver-related conditions, with liver cirrhosis and liver cancer accounting for 61.7% and 38.3%, respectively^2^. Thus, novel strategies are needed to understand the disease mechanisms, enable effective treatment, and to develop biohybrid artificial liver systems as artificial extracorporeal supportive devices for patients with liver failure.

Successful liver tissue engineering requires appropriate cells, extracellular matrix (ECM), and signaling molecules^3^. By using three-dimensional (3D) scaffolds, the natural environment of the cells can be mimicked to improve the growth, organization, and function of seeded cells. Human primary hepatocytes are an ideal cellular source for liver regeneration, as they account for 60–80% of the liver mass and have many important functions in the human body. Especially, cytochrome P450 enzymes found in hepatocytes are responsible for drug metabolism. Furthermore, hepatocytes are crucial for bile secretion and endocytic blood filtration and contribute to the maintenance of glucose homeostasis in the body^4^. However, the applicability of primary human hepatocytes for tissue engineering as well as drug testing is compromised by their limited availability, lack of proliferative capacity, and rapid loss of functions during in vitro culture^5^. In addition, the genetic metabolic mechanisms of animal models differ from those of humans, meaning that the results cannot be often transferred to clinical practice. Thus, hepatocytes of human origin are needed for experimental and therapeutic studies. Several approaches exist to mimic the native liver microenvironment^6^, including the use of decellularized liver scaffolds^7^, 3D bioprinted hydrogels^8^, and fiber-based 3D scaffolds^9^. Various parameters, such as porosity, material and chemical properties, as well as the 3D architecture of the scaffolds, play an important role in controlling cellular functions and behavior^10^. Ideal scaffolds should therefore facilitate cell attachment, enable the supply of nutrients to maintain cell viability and biodegrade over time^9^. Various techniques have been established to fabricate porous structures from biodegradable polymers, such as solvent extraction^18^, 3D printing^19^, and electrospinning^20^.

Poly-ε-caprolactone (PCL) is a biocompatible, biodegradable, non-toxic, thermoplastic polyester that can be used in electrospinning to produce 3D scaffolds^11^. Furthermore, its chemical properties and degradability with very low acidification, as well as its mechanical strength can be easily adjusted^12^. Due to its adaptability, PCL can be used for the engineering of soft or hard tissues by changing its molecular weight and degradation time^12^. The porous structure of PCL scaffolds allows the efficient supply of nutrients to the cells and enables the maintenance of the cells in the 3D constructs^13^. Furthermore, when transplanted, endothelial cells can infiltrate the porous PCL scaffolds to revascularize the scaffolds^14,15^ and ensure the viability of the cells in the 3D PCL scaffolds. Several studies have shown that the use of porous scaffolds produced by electrospinning has provided a significant contribution to liver tissue engineering, in terms of cell functionality demonstrated by albumin secretion, urea synthesis, and enzymatic activity^16–18^. Interestingly, it has been found that electrospun PCL mats can exhibit some similar responses to original liver tissue^19^, especially the growth rate of hepatocytes on PCL scaffolds was comparable to natural tissue scaffolds such as decellularized porcine liver ECM. Thus, PCL has the potential to mimic the ECM as a cell growth-supporting material.

The combination of fused filament fabrication (FFF) 3D printing with melt electrospinning for tissue engineering approaches has been introduced in recent years^20^. In this study, melt electrospinning was used for scaffold formation. The electrohydrodynamic jetting technology with electrical instabilities results in the generation of fibers with larger diameters compared with solution electrospinning^21^. Other advantages of melt electrospinning compared to solution electrospinning include simple equipment, no need for solvents, higher productivity, and the ability to fabricate various structures for cell culture scaffolds.

In recent years, the ability to reprogram human somatic cells into human induced pluripotent stem cells (hiPSCs) has opened up novel possibilities for regenerative medicine by enabling the generation of autologous desired cell types, such as neurons, cardiomyocytes, or hepatocytes^22^. By avoiding the use of vectors that can be inserted into the genome of somatic cells during  the reprogramming, footprint-free hiPSCs can be obtained. The exogenous delivery of self-replicating messenger RNA (srRNA) into somatic cells leads to the expression of the desired reprogramming factors under physiological conditions by the cells’ translational machinery in the cytosol^23^. Since srRNA does not need to enter the nucleus to be translated into proteins, the risk of genomic integration and insertional mutagenesis is prevented. The use of srRNA instead of conventional synthetic messenger RNA (mRNA) also eliminates the need for daily transfection of the cells until the reprogramming is complete^24^.

In this study, srRNA was used to obtain footprint-free hiPSCs from adult human epithelial cells derived from urine. A differentiation protocol was established to generate hepatocyte-like cells (HLCs) from these hiPSCs. The successful generation of HLCs was demonstrated by the expression of hepatocyte-specific markers at the transcriptional and protein levels. In addition, the obtained cells exhibited essential hepatic functions such as cytochrome P450 activity, albumin synthesis, indocyanine green metabolism, and glycogen storage. The use of melt-electrospun PCL scaffolds facilitated the 3D cultivation and differentiation of hiPSC-derived hepatoblasts into HLCs. These 3D constructs can be applied for disease modeling, drug screening, as well as for tissue reconstruction studies by using patient-specific HLCs.

## Materials and methods

### Ethics statement

Renal epithelial cells were isolated from the urine of adult healthy donors, which gave written informed consent to participate. The study was approved by the Ethics Committee of the Medical Faculty of the University of Tuebingen (911/2018BO2). All experiments were performed in accordance with relevant guidelines and regulations. Since no living animals were used in this study, ethical approval for animal testing was not required.

### Fabrication of melt-electrospun PCL scaffolds

The PCL (Internal labeling: batch number PCL-19) used for this study was produced by ITV Denkendorf Productservice GmbH. The inherent viscosity was 1.9 dl/g and the melt temperature 57.5 °C ± 2.5 °C. PCL filaments with 1.75 mm diameter (ITV Denkendorf Productservice GmbH) were produced using a fused filament fabrication 3D printing device (German RepRap X350pro, InnovatiQ GmbH + Co KG, Feldkirchen, Germany) which was modified using a high voltage electrostatic source (Eltex KNH34, Eltex-Elektrostatik GmbH, Weil am Rhein, Germany) and an aluminum printing bed as electrostatic target. PCL filaments were extruded through printing nozzles with a diameter of 0.1 mm and a nozzle spacing of 25 mm. A melt jet was then generated with an accelerating voltage that enabled the manufacturing of fibers with smaller diameters at 265 °C and a printing speed of 0.2 mm/s. With this process, 3D nonwoven PCL scaffolds were fabricated. The melt-electrospun PCL scaffolds were analyzed via scanning electron microscopy (SEM) imaging (Hitachi TM1000, Tokyo, Japan) and characterized via directional diameter analysis (MAVIfiber2d, Fraunhofer ITFM, Germany). The printing parameters were controlled by slicing software (Simplify3D, Cincinnati, USA).

### Swelling behavior analysis

The swelling capacity of scaffolds plays an important role in the absorption of body fluids and the transfer of nutrients and metabolites. Thus, swelling studies were performed to determine the water absorption capacity of the fabricated PCL scaffolds. The dry weight (W_d_) of the scaffold was determined before it was immersed in 1 ml of Dulbecco's phosphate-buffered saline (DPBS) for 24 h at 37 °C. After incubation, scaffolds were taken out of DPBS, excess DPBS on the surface was removed with filter paper, and their wet weight (W_w_) was determined. The swelling ratio was determined by (W_w_ − W_d_)/W_d_.

### Coating of PCL scaffolds with vitronectin

Scaffolds were incubated for 1 h at room temperature (RT) with 70% EtOH. Subsequently, the EtOH was evaporated overnight under the sterile bench. Scaffolds were placed in 12-well plates and coated with 0.5 ml of 5, 10, or 20 µg/ml vitronectin dissolved in DPBS for 1 h at RT. Afterwards, the vitronectin coating solution was aspirated.

### Cultivation of HepG2 cells

HepG2 cells (ECACC General Cell Collection, Porton Down, UK) were cultured in T75 flasks in Dulbecco's Modified Eagle Medium: Nutrient Mixture F-12 (DMEM/F-12 medium) (Gibco by Life Technologies, Carlsbad, CA, USA) supplemented with 10% FBS (Thermo Fisher Scientific). Medium changes were performed every 2–4 days. At a confluence of 80%, the cells were washed once with DPBS and detached with 0.05% trypsin–EDTA (Gibco by Thermo Fisher Scientific) for 5 min at 37 °C. Cells were then centrifuged for 5 min at 300×*g* and cultivated with a splitting ratio of 1:10 or 1:20.

### Seeding of HepG2 cells on vitronectin-coated PCL scaffolds

After vitronectin coating, the scaffolds were soaked in 200 µl HepG2 cell culture medium. 5 × 10^5^ HepG2 cells were resuspended in 100 µl HepG2 medium and the suspension was applied dropwise onto the scaffold and incubated for 1 h to allow the cells to attach to the scaffold. Afterwards, 1 ml fresh culture medium was added per well. After 24 h, cell attachment and morphology were analyzed.

### Cultivation of human renal epithelial cells (RECs) from human urine

RECs were isolated and cultivated as described in our recently published study^23^. Briefly, around 200 ml of urine from human healthy volunteers was collected. Approximately 20–50 RECs were obtained and cultivated at 37 °C with 5% CO_2_ in gelatin-coated well plates (Sigma-Aldrich, Steinheim, Germany) in REC proliferation medium, consisting of 50% renal epithelial basal medium (Lonza, Basel, Switzerland) in combination with 50% proliferation medium. Medium changes were performed every third day. At 80% confluency, RECs were used for further experiments.

### Reprogramming of RECs using srRNA into hiPSCs

Footprint-free hiPSCs were generated by reprogramming of RECs using VEE-OKSiM-GFP srRNA encoding OCT4, KLF4, SOX2, cMYC, and GFP. The transfection and reprogramming were performed according to our recently published study^23^.

### Cultivation of hiPSCs derived from RECs

The obtained hiPSCs were cultivated in Essential 8 medium (E8 stem cell medium, Thermo Fisher Scientific) on T25 culture flasks coated with 5 µg/ml vitronectin (Thermo Fisher Scientific) at 37 °C and 5% CO_2_. At 70% confluence, hiPSCs were detached with 0.5 mM EDTA (Sigma-Aldrich, Steinheim, Germany) for 5 min at 37 °C. Then, the cells were resuspended in E8 medium along with 10 µM Y-27632 (ROCK inhibitor, Enzo Life Sciences, Lausen, Switzerland) and seeded in 12-well plates for hepatic differentiation. The medium was changed after 24 h to E8 medium without ROCK inhibitor and daily medium changes were performed.

### Differentiation of hiPSCs towards HLCs

2 × 10^4^ hiPSCs (passage 25–40) were seeded in vitronectin-coated (5 µg/ml) 12-well plates and cultivated for 2 days at 37 °C and 5% CO_2_. The medium was changed daily (1 ml/well). To initiate the differentiation, E8 medium was changed to endoderm medium 1 (1 ml/well) containing 0.5% DMSO (SERVA Serving Scientists, Heidelberg, Germany) in E8 medium. After 24 h, the medium was changed to endoderm medium 2 containing RPMI 1640 (Thermo Fisher Scientific) with 3 μM CHIR99021 (Peprotech) and 1% B27 supplement minus insulin (Thermo Fisher Scientific). The next day, endoderm medium 3, RPMI 1640 medium containing 1% B27 supplement (Thermo Fisher Scientific) was applied for another 24 h.

To improve differentiation efficiency, undifferentiated hiPSCs were eliminated selectively using RPMI 1640 medium (Thermo Fisher Scientific) supplemented with 1.2 mol/l l-alanine (Thermo Fisher Scientific)^25^. Therefore, after endoderm induction (day 3), RPMI 1640 medium supplemented with L-alanine was added to the cells and incubated for 1 h at 37 °C. Then, the cells were cultivated for another 24 h with endoderm medium 3. Then, at day 4, a medium change was performed and the endodermal cells were cultivated in RPMI 1640 with 1% B27 supplement, 20 ng/ml bone morphogenic protein 4 (BMP4) (Peprotech), 5 ng/ml basic fibroblast growth factor (bFGF) (Peprotech) and 0.5% DMSO for another 5 days to induce hepatoblast stage. The culture medium was changed daily (1 ml/well). For the differentiation of hepatoblasts towards HLCs, the cells were first cultivated for 5 days in RPMI 1640 containing 0.5% DMSO, 1% B27 supplement, and 20 ng/ml hepatocyte growth factor (HGF) (Peprotech). The medium was changed daily (1.5 ml/well). Then, immature hepatocytes were further differentiated in hepatocyte culture medium BulletKit (Lonza, Basel, Switzerland) supplemented with 20 ng/ml HGF, 20 ng/ml Oncostatin M (OSM) (Peprotech), 100 nM dexamethasone and 0.5% DMSO for 7 days to obtain mature HLCs. The medium was changed every other day (2 ml/well).

As a negative control, 2 × 10^4^ hiPSCs (passage 25–40) were seeded in vitronectin-coated (5 µg/ml) 12-well plates and cultivated at 37 °C and 5% CO_2_ using E8 medium along with 10 µM Y-27632 ROCK inhibitor. The medium was changed after 24 h to E8 medium without ROCK inhibitor and daily medium changes were performed until the corresponding differentiation stage of HLCs was completed.

### Seeding of hiPSC-derived hepatoblasts on vitronectin-coated PCL scaffolds and differentiation into HLCs

On day 3 after inducing the hepatoblast differentiation of definitive endoderm cells (3 days after selection with L-alanine), the cells were detached using TrypLE Express (Gibco by Life Technologies) and 5 × 10^5^ hepatoblasts in 100 µl hepatoblast medium supplemented with 2% KnockOut Serum Replacement (KOSR) (Gibco by Life Technologies), 1% B27, and 10 µM ROCK inhibitor Y-27632 were seeded onto scaffolds coated with 20 µg/ml vitronectin. The cell suspension was added dropwise onto the scaffold and incubated for 1 h at 37 °C with 5% CO_2_ to let the cells attach to the scaffold. Afterwards, 1 ml of fresh hepatoblast culture medium was added per scaffold. After the colonization of the scaffolds, cultivation was performed in hepatoblast medium for an additional 2 days. Then, cells were differentiated into mature HLCs according to the described differentiation protocol above.

Simultaneously with the transfer of the hepatoblasts to the PCL scaffolds, 5 × 10^5^ hiPSCs (passage 25–40) were seeded in 100 µl E8 medium and 10 µM Y-27632 ROCK inhibitor onto scaffolds coated with 20 µg/ml vitronectin as a negative control. The cell suspension was added dropwise onto the scaffolds and incubated for 1 h at 37 °C with 5% CO_2_ to allow the cells to attach. Afterwards, 1 ml of fresh E8 medium with ROCK inhibitor was added to each scaffold. The medium was changed after 24 h to E8 medium without ROCK inhibitor and daily medium changes were performed.

### Uptake and release of indocyanine green (ICG) by hiPSC-derived HLCs

The metabolization of ICG (Diagnostic Green GmbH, Aschheim, Germany) was analyzed to evaluate the ability of the generated hiPSC-derived HLCs for the uptake, conjugation, and release of the substance. ICG can only be metabolized by functional hepatocytes and is also clinically established to detect the functionality of hepatocytes. For the preparation of a stock solution, ICG was dissolved in DMSO (5 mg/ml). Then, cells were incubated with 1 mg/ml ICG in 1 ml hepatocyte culture medium BulletKit for 30 min at 37 °C with 5% CO_2_. Cells were then washed 3× with DPBS and ICG uptake was analyzed by phase-contrast microscopy. Afterwards, the medium was replaced by fresh medium and the cells were incubated for another 6 h at 37 °C with 5% CO_2_ followed by microscopic examination. Images were acquired using an Axiovert135 microscope (Carl Zeiss, Oberkochen, Germany) and EOS Utility software (Canon, Tokyo, Japan).

### Detection of cytochrome P450 activity

To detect cytochrome P450 activity, hiPSC-derived HLCs differentiated in 12-well plates were washed with 1 ml DPBS. For cytochrome P450 induction, cells were incubated with 25 µM and 50 µM rifampicin (Merck, Darmstadt, Germany) in hepatocyte culture medium BulletKit for 48 h. Afterwards, the cells were washed once with 1 ml DPBS. The cytochrome P450 activity was analyzed using a nonlytic P450-Glo assay (Luciferin-IPA) (Promega, Madison, USA) according to the manufacturer’s protocol. Therefore, cells were incubated with CYP3A4/Luciferin-IPA diluted 1:1000 in hepatocyte culture medium BulletKit (Lonza, Basel, Switzerland) at 37 °C with 5% CO_2_ for 45 min. Next, 25 µl of the supernatant was transferred into one well of a white 96 well-plate, and 25 µl luciferin detection reagent was added. The CYP3A4 activity was then measured in triplicates with a fluorescence microplate reader (Mithras LB 940, Berthold Technologies, Bad Wildbad, Germany).

### Flow cytometry analysis

Cells were washed and detached using TrypLE Express (Gibco by Life Technologies). The cells were then centrifuged for 3 min at 600×*g* and washed with 1 ml DPBS. For intracellular staining, cells were fixed for 15 min with 4% paraformaldehyde (PFA) at RT. After a washing step with DPBS, cells were suspended in permeabilization buffer (2% BSA in DPBS and 0.2% Triton X-100), and fluorescently labeled antibodies were added at a concentration indicated by the manufacturer and incubated at RT for 45 min. For extracellular staining, cells were suspended in wash buffer (2% BSA in DPBS) and fluorescently labeled antibodies were added at a concentration indicated by the manufacturer and the sample was incubated for 45 min at RT. Following this, a washing step was performed, then the cells were suspended in 500 µL CellFIX (1×) (Becton Dickinson, Heidelberg, Germany) and analyzed via flow cytometry analysis using the following antibodies: PE-labeled mouse anti-human CXCR4 and PE-labeled mouse anti-human alpha-fetoprotein antibodies (both from R&D Systems, Minneapolis, USA), PE-labeled mouse anti-human FOXA2 and PE-labeled mouse anti-human ASGPR1 antibodies (both from Miltenyi Biotec, Bergisch Gladbach, Germany), Alexa Fluor 488-labeled goat anti-human ALB antibody (Thermo Fisher Scientific) and DyLight 488-labeled mouse anti-human TRA-1–60 antibody (Stemgent, Cambridge, USA).

### qRT-PCR

RNA was isolated with Aurum™ Total RNA Mini Kit (Bio-Rad, Munich, Germany). Using the iScript kit (Bio-Rad, Hercules, USA) 300 ng RNA from cells was then reverse-transcribed into cDNA. Primers (final concentration: 300 nM) used are listed in Table 1. They were obtained from Eurofins Genomics (Ebersberg, Germany). IQ SYBR Green Supermix (Bio-Rad) and the CFX Connect Real-Time PCR Detection System (Bio-Rad) were used to perform qRT-PCR analyses. GAPDH (glyceraldehyde 3-phosphate dehydrogenase) was taken as housekeeping gene. Primers were self-designed with the Primer-Blast tool from NCBI^26^. To control melting temperatures and self-complementarities, the Oligonucleotide Properties Calculator from Northwestern University Medical School was used^27^.

**Table 1:** List of all primer sequences, used for gene expression studies.

Marker for	Gene	Sense primer 5′–3′	Antisense primer 5′–3′
–	GAPDH	TCAACAGCGACACCCACTCC	TGAGGTCCACCACCCTGTTG
Stem cells	Nanog	TGAACCTCAGCTACAAACAG	TGGTGGTAGGAAGAGTAAAG
Endoderm	CXCR4	TCCATTCCTTTGCCTCTTTTGC	TGTCCGTCATGCTTCTCAGTT
	SOX17	GATTGCACTGGTCACCTCGG	TCCGTGTAATAAGGGTCTTCGC
	FOXA2	TGCACTCGGCTTCCAGTATG	CGTGTTCATGCCGTTCATCC
Hepatoblasts	HNF4α	ACTACATCAACGACCGCCAGT	ATCTGCCAGGTGATCCTCTG
	AFP	AAATGCGTTTCTCGTTGCTT	GAGTTGGCAACAAGTGGCTG
HLCs	ALB	GCACAGAATCCTTGGTGAACAG	ATGGAAGGTGAATGTTTCAGCA
	CYP3A4	CCGAGTGGATTTCCTTCAGCTG	TGCTCGTGGTTTCATAGCCAGC
	CYP2C9	CAAGATTTTGAGCAGCCCCTG	TGGTTGTGCTTTTCCTTCTCCA
	APOA2	GCCGAGGCCAAGTCTTACTTT	GCTGTGTTCCAAGTTCCACG
	A1AT	AGGTGCCTATGATGAAGCGT	TCAGGCAGGAAGAAGATGGC
	CYP1A2	ATGTGAGCAAGGAGGCTAAGG	GGCAGTCTCCACGAACTCA
	CYP2D6	GGTGGTCGTGCTCAATGGG	GCGAAAGGGGCGTCCTTG

*GAPDH* glyceraldehyde-3-phosphate dehydrogenase, *Nanog* homeobox protein NANOG, *CXCR4* C–X–C chemokine receptor type 4, *SOX17* SRY-box 17, *FOXA2* forkhead box protein A2, *HNF4α* hepatocyte nuclear factor 4 alpha, *AFP* alpha-fetoprotein, *ALB* albumin, *CYP3A4* cytochrome P450 3A4, *CYP2C9* cytochrome P450 2C9, *CYP1A2* cytochrome P450 1A2, *CYP2D6* cytochrome P450 2D6, *APOA2* apolipoprotein A-II, *A1AT* alpha-1 antitrypsin.

### Immunofluorescence staining

After washing with DPBS, the cells were fixed for 15 min with 4% PFA at RT. Then, cells were blocked for 45 min at RT in 4% BSA. For intracellular staining, permeabilization buffer (DPBS containing 2% BSA and 0.2% Triton X-100) with fluorescently labeled antibodies at concentrations indicated by the manufacturer was applied for 1 h at RT. For extracellular staining, cells were incubated in washing buffer (2% BSA in DPBS) with fluorescently labeled antibodies. The cells were then washed 3× with permeabilization buffer or washing buffer and 1× with DPBS. If a secondary antibody was used, cells were treated for 1 h at RT with permeabilization or washing buffer and the secondary antibody. Subsequently, the cells were rinsed 3× with permeabilization buffer or washing buffer and then 1× with DPBS. Finally, the cells were covered with DAPI mounting medium (Abcam, Cambridge, UK). The following antibodies were used: PE-labeled mouse anti-human CXCR4 and PE-labeled mouse anti-human alpha-fetoprotein antibodies (both from R&D Systems), PE-labeled mouse anti-human FOXA2 and PE-labeled mouse anti-human ASGPR1 antibodies (both from Miltenyi Biotec), and Alexa Fluor 488-labeled goat anti-human albumin antibody (Thermo Fisher Scientific). Using a fluorescence microscope (Axiovert 135 microscope with AxioVision 4.8.2 software from Carl Zeiss), images were acquired.

### F-actin staining of cells

To visualize the attachment and distribution of cells on the scaffolds, F-actin staining was performed using ActinRed™ 555 (Thermo Fisher Scientific, Waltham, MA, USA). Therefore, scaffolds seeded with cells were washed twice with DPBS and fixed with 4% PFA at RT for 15 min. Subsequently, the scaffolds were washed again with DPBS and incubated for 15 min at RT in 1 ml permeabilization buffer containing 2% BSA, 0.2% Triton X-100, and 1 drop of ActinRed™ 555. In the end, the scaffolds were stained with DAPI mounting medium (Abcam) to visualize the cell nuclei.

### Periodic acid-Schiff (PAS) staining for the detection of glycogen synthesis

To identify glycogen storage in hiPSC-derived HLCs, PAS staining was performed at the end of the differentiation process (day 20) using a PAS staining kit (Morphisto GmbH, Offenbach am Main, Germany). Therefore, cells were washed 2× with DPBS and fixed with 4% PFA at RT for 15 min. Subsequently, the cells were treated with 1% periodic acid solution at RT for 10 min and then incubated in Schiff’s reagent at RT for 15 min. After rinsing with water, cells were stained with Mayer’s hematoxylin for 5 min at RT. Images were acquired using an Axiovert135 microscope (Carl Zeiss) and EOS Utility software (Canon).

### Detection of albumin secretion by enzyme-linked immunosorbent assay (ELISA)

At the end of the hepatocyte differentiation process (day 20), the HLCs were cultivated for 24 h with 2 ml medium. To detect secreted albumin in the supernatant of the generated HLCs, albumin concentration in 100 µl was determined in duplicate using a human albumin-specific ELISA (Thermo Fisher) as described by the manufacturer. The absorbance was measured at 450 nm using a microplate reader (BioTek Instruments, Winooski, USA).

### Statistical analysis

Data are presented as mean ± SEM or SD. For data analysis of repeated measurements one-way ANOVA, paired or unpaired t-test followed by Bonferroni’s multiple comparison test was applied. Statistical analyses were performed using GraphPad Prism 7.00 (GraphPad Software, La Jolla, CA, USA). Differences of p < 0.05 were considered significant.

## Results

### Differentiation of footprint-free generated hiPSCs into HLCs

**Figure 1:**
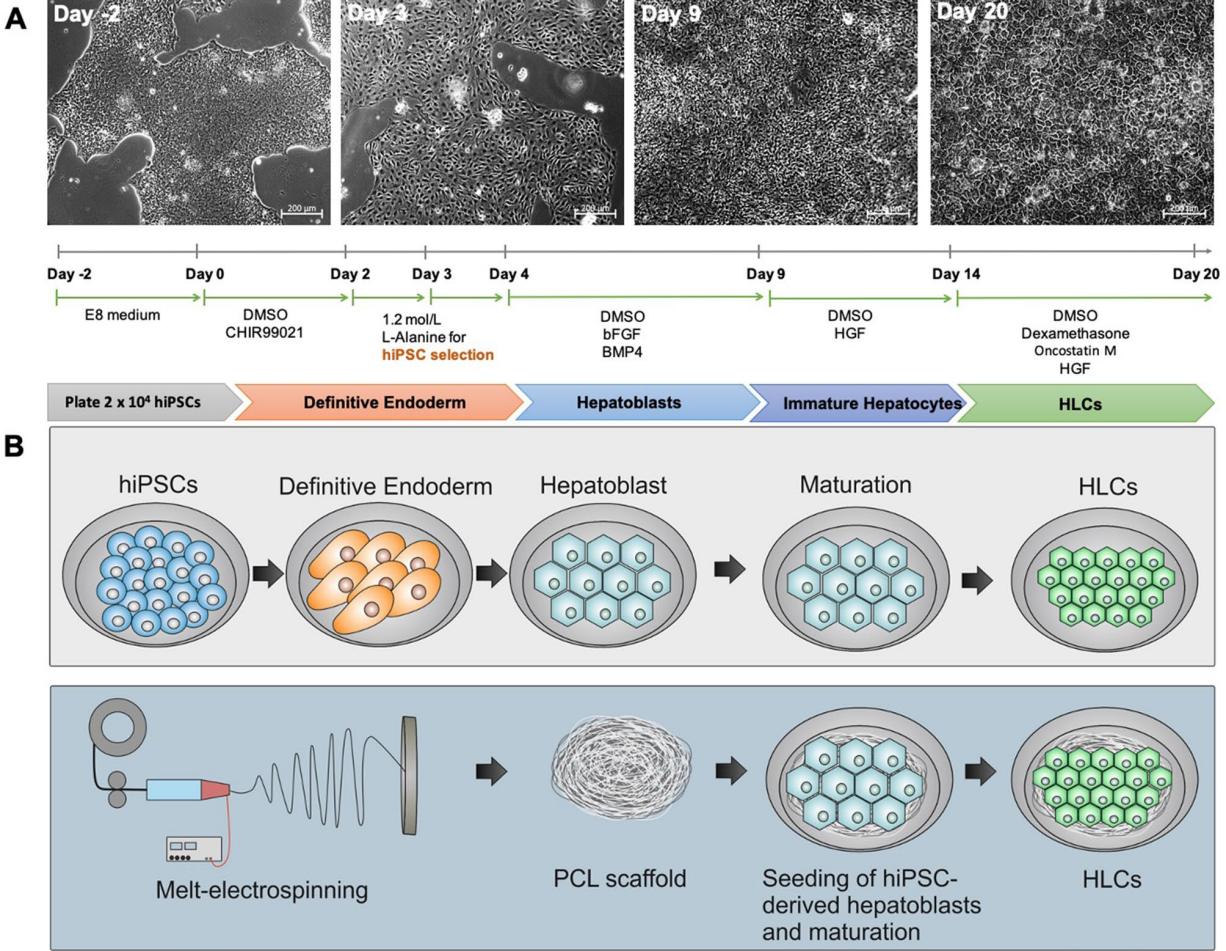
Differentiation of hiPSCs into HLCs and cultivation on 3D melt-electrospun poly-ε-caprolactone (PCL) scaffolds. **(A)** The sequence of the protocol for the differentiation of human renal epithelial cells derived hiPSCs into HLCs and morphological overview of cells at different stages. **(B)** Schematic representation of the hepatic differentiation, the fabrication of 3D melt-electrospun PCL scaffolds, the following population with hiPSC-derived hepatoblasts and their maturation towards HLCs within the three-dimensional (3D) construct.

After reprogramming adult somatic RECs into hiPSCs, the cells were seeded into vitronectin-coated wells of a 12-well plate to induce hepatic differentiation. A 20-day protocol using a cocktail of small molecules and growth factors was applied. The protocol was divided into 4 differentiation stages of the cells including definitive endoderm, hepatoblasts, immature hepatocytes, and mature HLCs (Fig. 1A). After 3 days of endoderm differentiation, hiPSC colonies transformed into flatter cells with prominent nuclei similar to early hepatoblasts. At this point of differentiation, a selective elimination of undifferentiated hiPSCs was established by incubation of the cells with l-alanine for 1 h. After this selection, the cells underwent a continuous morphological change. After completion of endodermal induction and subsequent L-alanine selection, hepatic differentiation was initiated on day 4, and the cells transformed from clusters to hepatoblast-specific cuboidal shapes. At the end of the maturation phase, the generated HLCs showed hepatocyte-specific polygonal morphology.

To allow hepatocyte differentiation and growth in a 3D environment, 3 days after starting the hepatoblast differentiation of definitive endoderm cells, 5 × 10^5^ hiPSC-derived hepatoblasts were seeded onto vitronectin-coated PCL melt-electrospun scaffolds (Fig. 1B) and cultivated for 2 days until hepatoblast differentiation was complete. Cells were either analyzed or further differentiated into HLCs within the scaffold for 12 days.

### Induction of definitive endoderm stage and subsequent elimination of undifferentiated hiPSCs using l-alanine

**Figure 2:**
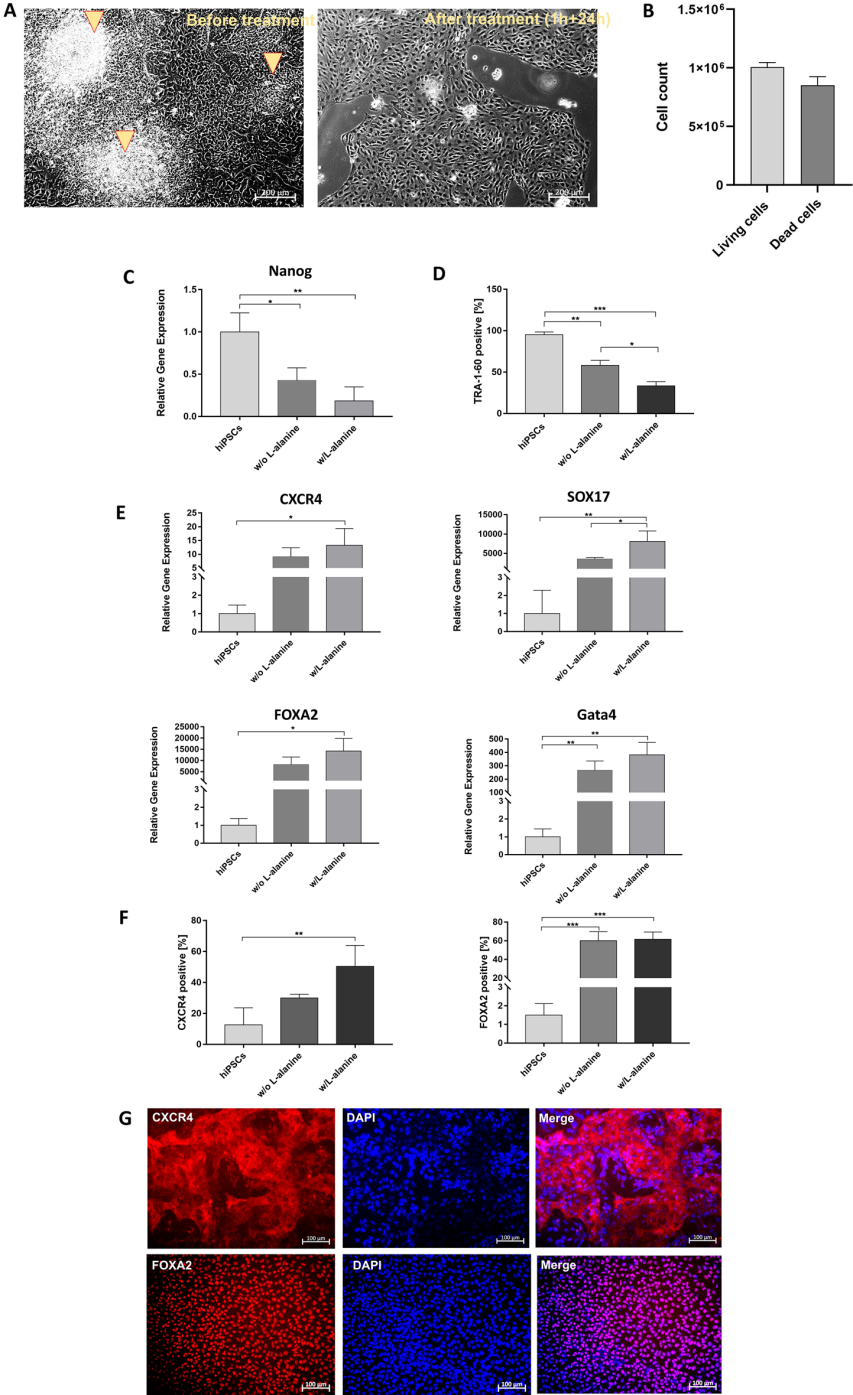
Elimination of undifferentiated hiPSCs with l-alanine treatment and analysis of endoderm differentiation. (**A**) Microscopic images of endoderm cells before and after l-alanine treatment for 1 h. Arrows show undifferentiated hiPSCs before selection. Scale bars show 200 µm. (**B**) Determination of living and dead cells 24 h after l-alanine treatment using hemocytometer. The results are presented as mean + SEM (n = 3). (**C**) qRT-PCR analysis of the presence of hiPSC-specific Nanog expression. mRNA levels were normalized to GAPDH, and the results are shown relative to hiPSCs. (**D**) Flow cytometry analysis of TRA-1–60-expressing cells after endoderm induction and selective elimination of undifferentiated hiPSCs. (**E**) Expression analysis of CXCR4, SOX17, FOXA2, and Gata4 transcripts performing qRT-PCR. (**F**) Flow cytometry analysis of CXCR4 and FOXA2-expressing cells after endoderm induction and selective elimination of undifferentiated hiPSCs. (**G**) Representative immunofluorescence microscopy images of endodermal cells after selection, stained with CXCR4 and FOXA2-specific antibodies. Scale bars represent 100 µm. All results are presented as mean + SEM (n = 3). Statistical differences were identified with one-way ANOVA (*p < 0.05; **p < 0.01; ***p < 0.001).

After induction of the definitive endoderm stage using DMSO and CHIR99021, cells were incubated for 1 h with l-alanine to eliminate undifferentiated hiPSCs and then cultivated for 24 h in endoderm medium 3. The treatment of cells for 1 h with l-alanine resulted in the successful reduction of residual hiPSCs (Fig. 2A). Phase-contrast microscopy analyses showed the elimination of residual hiPSC clusters, which resulted in pure definitive endoderm cell populations compared with the unselected cells.

24 h after the l-alanine treatment, approximately 8.5 × 10^5^ cells per well were dead and 1 × 10^6^ cells per well were viable (Fig. 2B). Furthermore, qRT-PCR revealed a significant reduction of the stem cell marker expression (Nanog) in endoderm cells compared with the initial hiPSCs (Fig. 2C). Moreover, flow cytometry analyses confirmed a significant reduction of TRA-1–60 expressing cells from 58.1 ± 8.4 to 33.4 ± 7.1% TRA-1–60 expressing cells after l-alanine selection (Fig. 2D).

The efficiency of endoderm differentiation was determined by the analysis of the endoderm specific marker expression levels of CXCR4, FOXA2, SOX17, and Gata4 by qRT-PCR with and without l-alanine treatment (Fig. 2E). Compared to hiPSCs, significantly increased expression of all analyzed genes was detected after the treatment of cells with l-alanine, and successful endoderm induction was demonstrated by an upregulation of the expression levels detected in endoderm cells compared to the initial hiPSCs. Flow cytometry analyses showed an increase in CXCR4 and FOXA2 positive cells after the l-alanine treatment (Fig. 2F). Furthermore, the increased level of CXCR4 and FOXA2 positive cells compared to the initial hiPSCs showed successful endoderm induction. The fluorescence microscopy analyses proved also the presence of CXCR4 and FOXA2 expressing cells (Fig. 2G) within the endoderm cells after 1 h of treatment. Since the analyses demonstrated that the l-alanine treatment effectively eliminates undifferentiated hiPSCs, the following experiments were performed with cells treated for 1 h with l-alanine.

## Characterization of generated hiPSC-derived hepatoblasts

**Figure 3:**
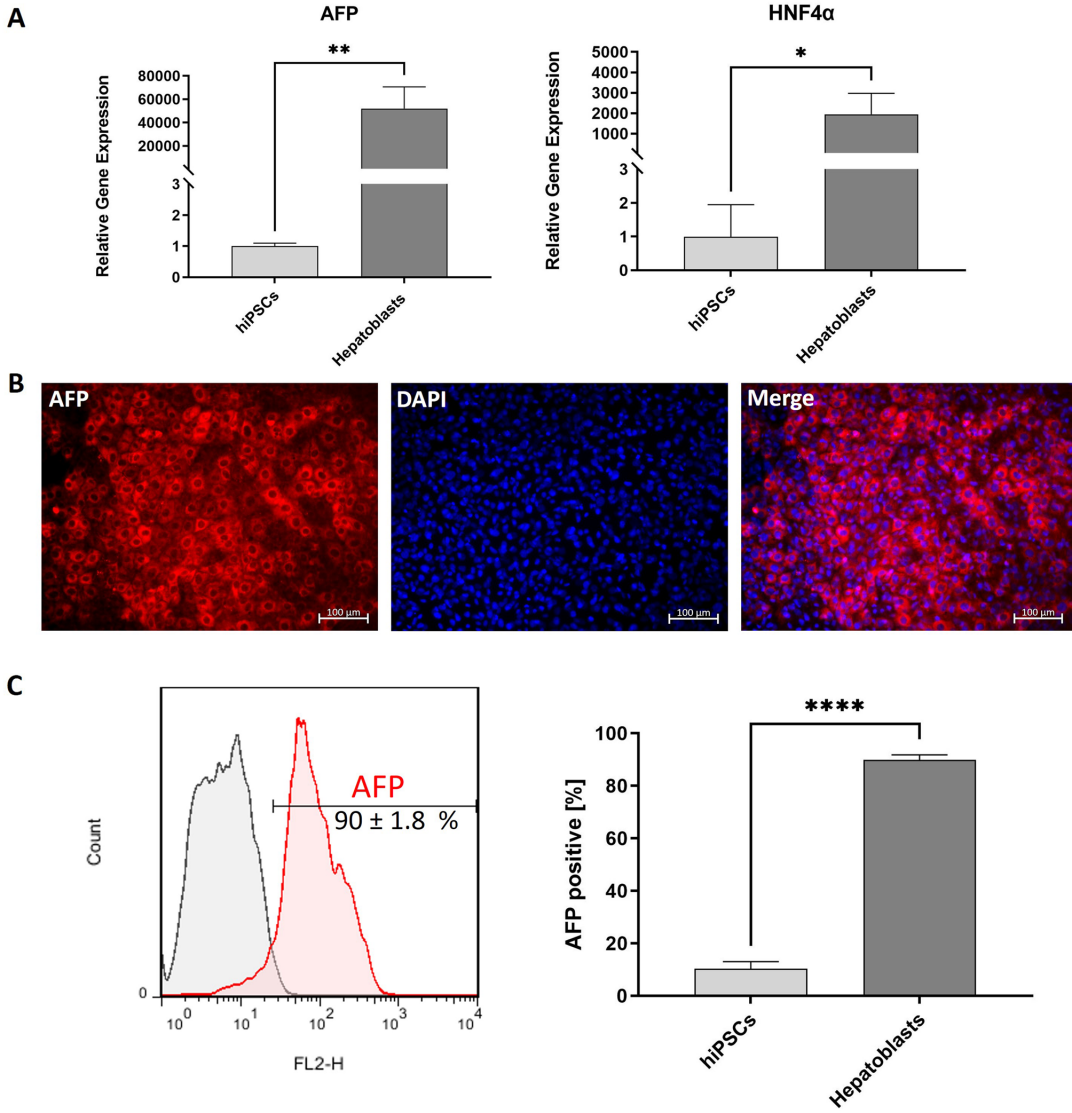
Characterization of generated hepatoblasts. (**A**) Expression analysis of AFP and HNF4α transcripts performing qRT-PCR. (**B**) Representative immunofluorescence microscopy images of hepatoblasts, stained with AFP-specific antibody (red) and nuclei with DAPI (blue). Scale bars represent 100 µm. (**C**) Flow cytometry analyses of AFP expression in cells after hepatoblasts differentiation. All results are presented as mean + SEM (n = 3). Statistical differences were identified using unpaired t-test (*p < 0.05; **p < 0.01; ****p < 0.0001).

After completion of the definitive endoderm stage including selective elimination of residual hiPSCs, the second stage of differentiation (hepatoblast stage) started at day 4. After 5 days of hepatoblast differentiation (day 9 in total), the expression levels of the specific hepatoblast markers alpha-fetoprotein (AFP) and hepatocyte nuclear factor 4 alpha (HNF4α) were analyzed. The qRT-PCR analysis at day 9 of the hepatic differentiation of hiPSCs-derived hepatoblasts confirmed the strong expression of HNF4α and AFP (Fig. 3A). The obtained hepatoblasts showed significantly higher expression of HNF4α (1945-fold) and AFP (51,938-fold) compared with the initial hiPSCs. The presence of AFP was also detected by immunostaining (Fig. 3B) of hiPSC-derived hepatoblasts. Flow cytometry analyses demonstrated that approximately 90% of the analyzed cells express AFP (Fig. 3C).

### Characterization and functionality analyses of the generated HLCs

**Figure 4:**
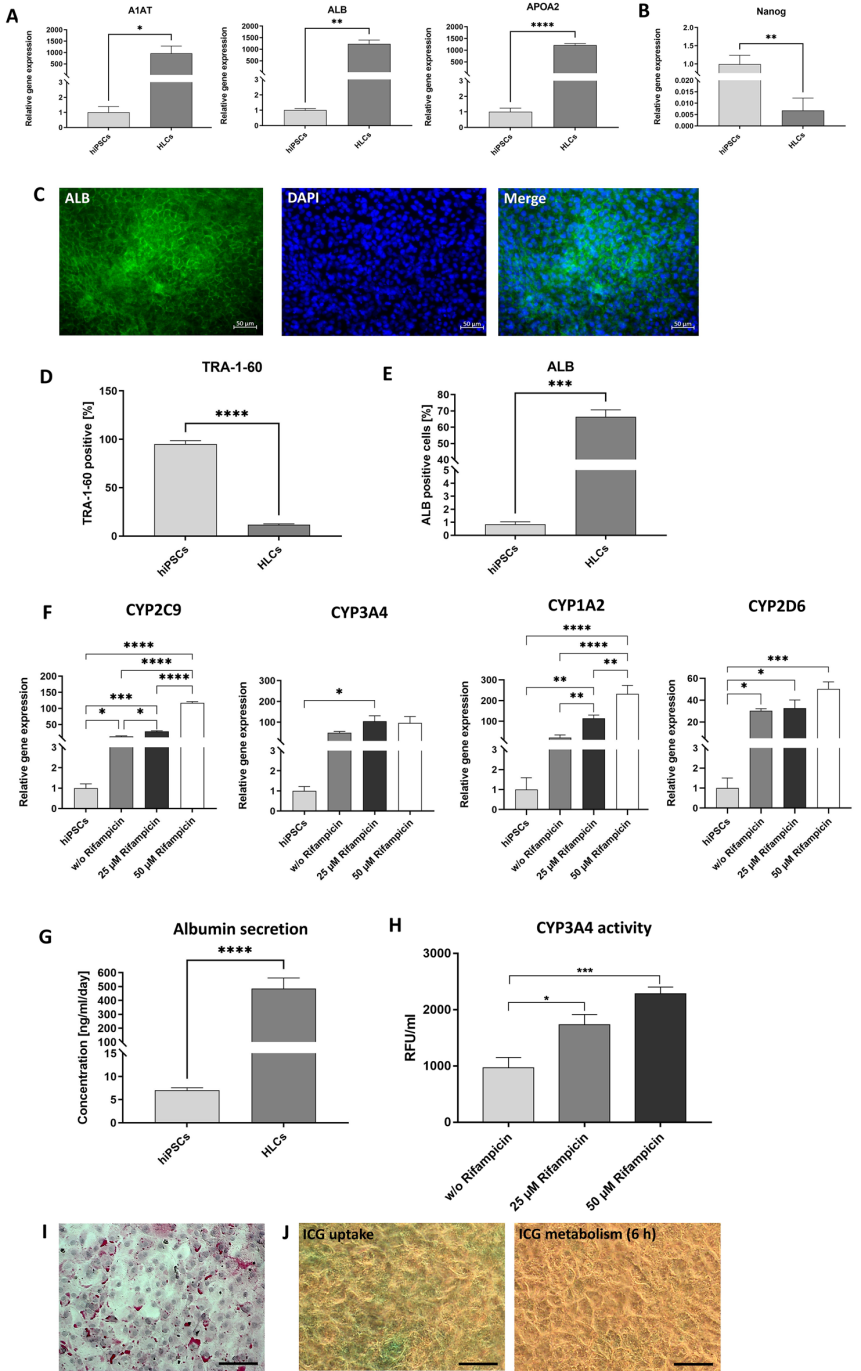
Hepatic maturation under 2D conditions and subsequent analyses of the cells. (**A**) Expression analysis of A1AT, ALB, and APOA2 transcripts performing qRT-PCR. mRNA levels were normalized to GAPDH, and the results are shown relative to hiPSCs (n = 3). (**B**) Expression analysis for the presence of the hiPSC marker Nanog. mRNA levels were normalized to GAPDH, and the results are shown relative to hiPSCs (n = 5). (**C**) Representative immunofluorescence microscopy images of HLCs, stained with albumin-specific antibodies. Scale bars show 50 µm or 100 µm. (**D**) Flow cytometry analyses of TRA-1–60 expressing cells after 20 days. All results are presented as mean + SEM (n = 3). Statistical differences were identified using unpaired t-test (****p < 0.0001). (**E**) Flow cytometry analyses of ALB-expressing cells after 20 days. All results are presented as mean + SEM (n = 3). Statistical differences were identified using unpaired t-test (***p < 0.001). (**F**) Expression analysis of CYP3A4, CYP2C9, CYP1A2, and CYP2D6 transcripts using qRT-PCR (n = 3). Expression levels were examined after stimulation with 25 µM and 50 µM rifampicin for 48 h and without rifampicin treatment. (**G**) Analysis of albumin secretion by ELISA (n = 5). Results were compared to the initial hiPSCs. (**H**) Metabolic activity of hiPSC-derived HLCs was determined by measuring the activity of cytochrome P450 CYP3A4 (n = 4). (**I**) PAS staining showing glycogen storage in hiPSC-derived HLCs. (**J**) Detection of ICG uptake (left) and release after 6 h. Scale bars represent 50 µm. All results are shown as mean + SEM. Statistical differences were identified with unpaired t-test or one-way ANOVA. (*p < 0.05; **p < 0.01; ***p < 0.001; ****p < 0.0001).

At day 20, the expression of hepatocyte-specific markers was evaluated. Gene expression analysis by qRT-PCR revealed significantly upregulated levels of ALB, APOA2, and A1AT expression compared to the initial hiPSCs (Fig. 4A). Furthermore, a significant reduction of the stem cell marker expression (Nanog) was detected by qRT-PCR in hiPSC-derived HLCs compared with the initial hiPSCs (Fig. 4B)**.** The presence of albumin could be confirmed by immunostaining (Fig. 4C). Moreover, flow cytometry analyses confirmed a significant reduction of TRA-1–60 expressing cells (Fig. 4D) and a significant increase of ALB expressing cells at day 20 (Fig. 4E). In addition, the expression of ASGPR1, HFE, and CD81 was examined, and a clear presence of each marker was detected (Supplementary Fig. 1). Furthermore, the functionality of the hiPSC-derived HLCs was analyzed. To examine metabolic activity, hiPSC-derived HLCs were treated with 25 µM and 50 µM rifampicin for 48 h, which resulted in increased CYP2C9 and CYP1A2 expression levels compared to unstimulated HLCs and initial hiPSCs (Fig. 4F). Moreover, a significant increase in CYP3A4 activity was measured after the treatment of cells with 25 µM and 50 µM rifampicin (Fig. 4H). In addition, significantly increased albumin secretion into the supernatant was detected in hiPSC-derived HLCs compared with the hiPSC control (Fig. 4G). PAS staining revealed the ability of the generated hiPSC-derived HLCs to store glycogen, as demonstrated by the pink staining of the cells (Fig. 4I). Another hepatocyte-specific function, the metabolization of ICG, was successfully demonstrated by uptake of the ICG (cells appeared green) and metabolization within 6 h (Fig. 4J**).**

### Scaffold fabrication and analysis of cell attachment to vitronectin-coated scaffolds

**Figure 5:**
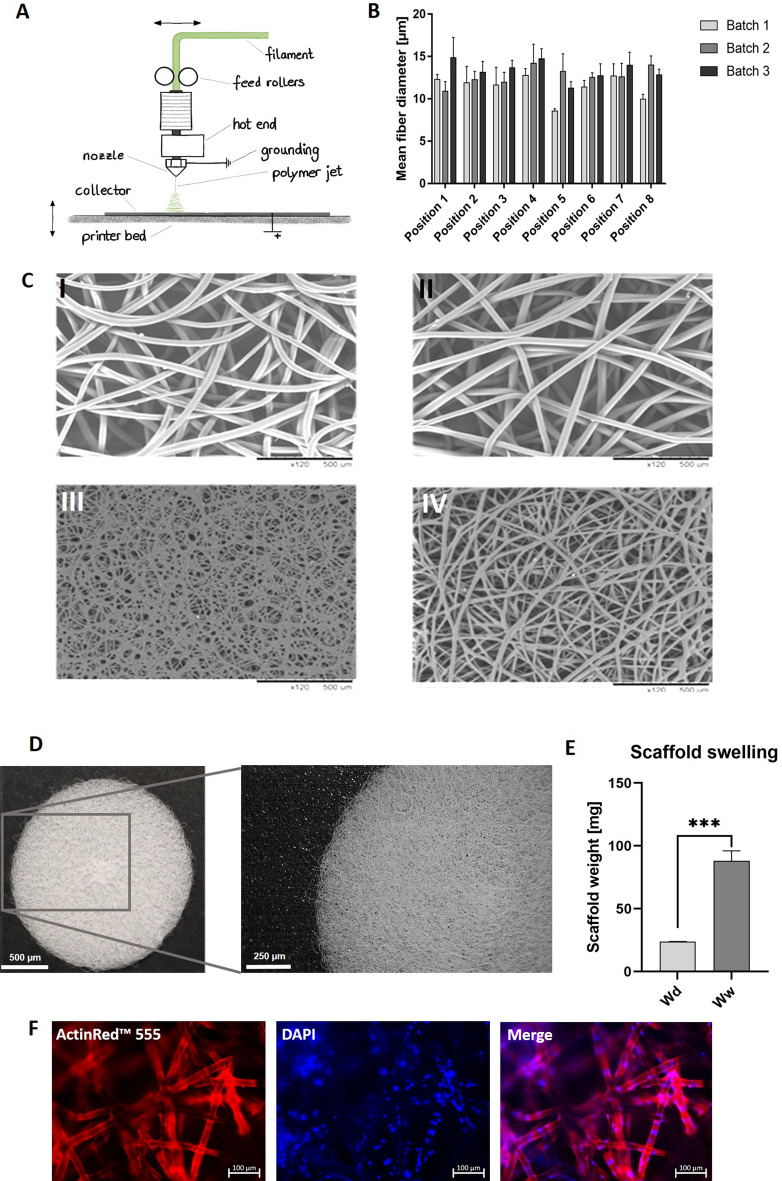
Fabrication of PCL scaffold and coating with vitronectin. (**A**) Schematic representation of PCL scaffold production using the extruder of a 3D printer and a high-voltage source. (**B**) The fiber diameters of the scaffold were analyzed at 8 different positions of the printing bed (n = 3). Scaffolds were fabricated with a printing speed of 0.2 mm/s, and an extrusion temperature of 265 °C. (**C**) Representative scanning electron microscopy (SEM) images of melt-electro-spun nonwoven scaffolds with varied printing speeds (I) 0.2 mm/s and (II) 0.4 mm/s and extrusion temperatures (III) 270 °C and (IV) 265 °C and a printing speed of 0.2 mm/s. The nozzle offset (25 mm) and the voltage (+ 15 kV) were kept constant. (**D**) Representative macroscopic images of PCL scaffold produced with the final setup. The scaffold had a diameter of 2 cm and fitted into a 12-well plate. Scale bars represent 500 µm and 250 µm. (**E**) Evaluation of the swelling behavior of PCL scaffolds. The dry weight (Wd) of the scaffold was determined before the scaffolds were immersed in DPBS for 24 h. After incubation, the wet weight (Ww) of the scaffolds was measured. (**F**) Representative immunofluorescence images of 5 × 10^5^ HepG2 cells seeded on 20 µg/ml vitronectin-coated PCL scaffolds and stained with ActinRed™ 555. Scale bars represent 100 µm.

The melt-electrospun PCL scaffolds were produced using an FFF 3D printing device combined with a high-voltage electrostatic source and an aluminum printing bed as the electrostatic target. Thereby, nonwoven scaffolds were printed on the printing bed (Fig. 5A). Two different printing speeds were analyzed, resulting in different scaffold structures. Printing with 0.2 mm/s resulted in scaffold structures with smaller pores (Fig. 5C I) compared to a printing speed of 0.4 mm/s (Fig. 5C II). Furthermore, two extrusion temperatures with a printing speed of 0.2 mm/s were tested. The application of 270 °C resulted in fused fibers (Fig. 5C III) that did not meet the requirements. The application of 265 °C resulted in uniform distribution and separation of the fibers (Fig. 5C IV). For the final setup of the scaffold fabrication, the voltage was kept constant at + 15 kV and a nozzle offset of 25 mm, a printing speed of 0.2 mm/s, and an extrusion temperature of 265 °C were used. Afterwards, the fiber diameter distribution from three scaffold batches was analyzed at eight different positions of the printing bed and resulted in a mean fiber diameter of 12.5 ± 1.4 µm (Fig. 5B). Only minor variations in fiber thickness between the batches were measured, which did not influence cell attachment. Round and porous scaffolds with a diameter of 2 cm were fabricated, which fitted into one well of a 12-well plate (Fig. 5D). After 24 h of incubation in DPBS, a swelling ratio of 2.69 ± 0.27 was determined, which showed the swelling behavior of the scaffolds. This was accompanied by an increase in the weight of the scaffolds (Fig. 5E).

To improve the adhesion of cells to the PCL fibers, the fibers were coated with different concentrations of vitronectin (5, 10, and 20 µg/ml) (Supplementary Fig. 2) and seeded with HepG2 cells. Coating with 20 µg/ml vitronectin (Fig. 5F) resulted in an even distribution and adhesion of cells around the fibers, 24 h after seeding of 5 × 10^5^ HepG2 cells onto the scaffold.

### Analysis of hepatoblasts seeded on PCL scaffolds

**Figure 6:**
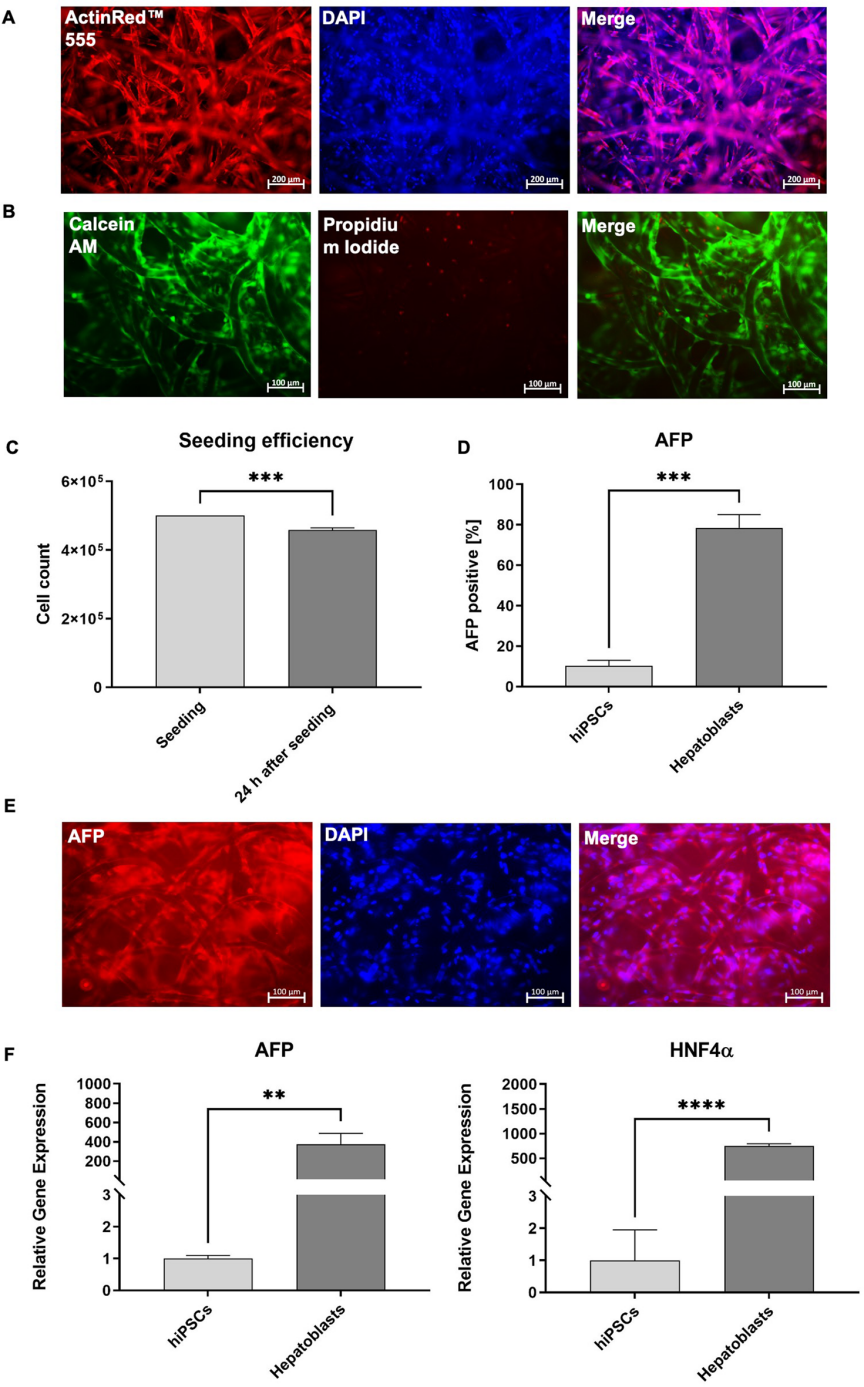
Analysis of hepatoblasts after the seeding on PCL scaffolds. (**A**) Analysis of the cell attachment and cell distribution within the scaffold using F-actin staining. (**B**) Live/dead staining of hepatoblasts, 24 after seeding on PCL scaffolds. Scale bars represent 100 µm. (**C**) Analysis of the seeding efficiency of hepatoblasts on PCL scaffolds 24 h after seeding of hepatoblasts on PCL scaffolds. (**D**) Flow cytometry analysis of AFP-expressing cells compared to the initial hiPSCs. (**E**) Representative immunofluorescence microscopy images of hepatoblasts, stained with AFP-specific antibodies. Scale bars represent 100 µm. (**F**) Expression analysis of AFP and HNF4α transcripts using qRT-PCR. mRNA levels were normalized to GAPDH, and the results are shown relative to hiPSCs. All results are presented as mean + SEM (n = 3). Statistical differences were identified with unpaired t-test (**p < 0.01; ***p < 0.001; ****p < 0.0001).

The coating of PCL scaffolds with 10 µg/ml vitronectin also resulted in less attachment of hepatoblasts (Supplementary Fig. 3) than with 20 µg/ml vitronectin coating, similar to the results with HepG2 cells. Thus, to perform the differentiation of hepatoblasts on PCL scaffolds, 5 × 10^5^ pre-differentiated hepatoblasts (at day 3 of hepatoblast differentiation) were seeded on 20 µg/ml vitronectin-coated scaffolds. Uniform attachment of the cells to the fibers and uniform distribution within the scaffolds were observed (Fig. 6A). At the end of hepatoblast differentiation within the PCL (3 days after seeding), live-dead staining was performed. Only a few dead cells were detected (Fig. 6B), indicating that the scaffold had no cytotoxic effect on the cells. The number of hepatoblasts attached to the fibers of the PCL scaffolds was determined 24 h after seeding (Fig. 6C) and showed that approximately 4.6 × 10^5^ cells were attached to the scaffold.

Flow cytometry analysis showed significantly higher numbers of cells expressing AFP (73.2 ± 6.9%) compared to the initial hiPSCs (Fig. 6D). The immunostaining of the cells 3 days after seeding demonstrated also a strong expression of the hepatoblast marker AFP (Fig. 6E). The qRT-PCR analysis revealed significantly higher expression levels of HNF4α and AFP, compared with the initial hiPSCs (Fig. 6F).

### Analysis of the maturation of hiPSC-derived hepatoblasts in PCL scaffolds into HLCs

**Figure 7:**
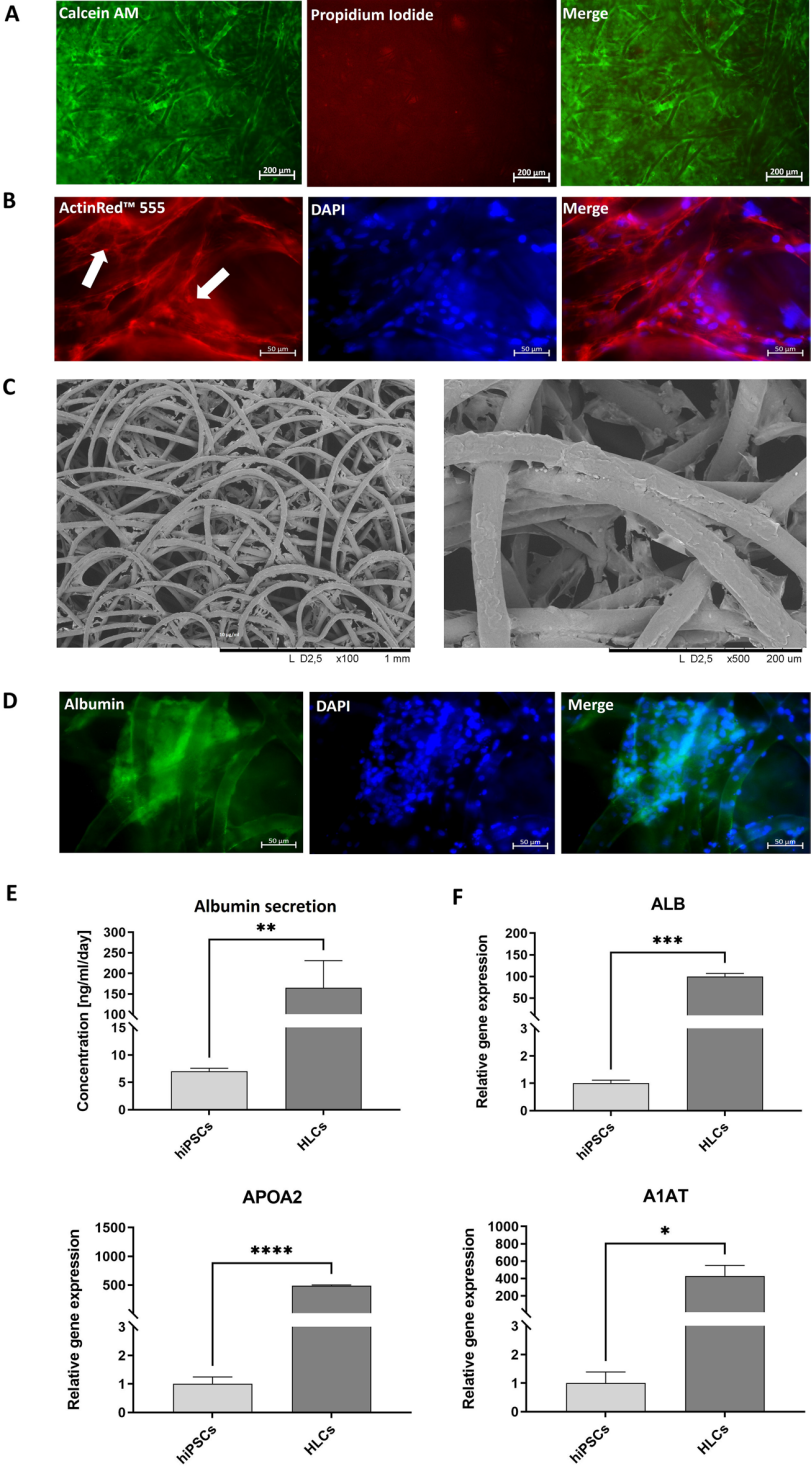
Analyses of PCL scaffolds after hepatic maturation and characterization of HLCs. (**A**) Live/dead staining of HLCs generated on PCL scaffolds. Scale bars represent 200 µm. (**B**) Analysis of the distribution and attachment of cells by F-actin staining. Cells attached to the scaffold and formed networks between the fibers (white arrows). Scale bars represent 50 µm. (**C**) Representative scanning electron microscopy (SEM) images of scaffolds containing hiPSC-derived HLCs. (**D**) Representative immunofluorescence microscopy images of HLCs stained with an albumin-specific antibody. Scale bars represent 50 µm. (**E**) Analysis of albumin secretion from generated HLCs compared with the initial hiPSCs using ELISA. Results are presented as mean + SEM (n = 5). Statistical differences were identified using unpaired t-test (**p < 0.01). (**F**) Expression analysis of ALB,APOA2, and A1AT transcripts by qRT-PCR. mRNA levels were normalized to GAPDH, and the results are shown relative to hiPSCs. Results are shown as mean + SEM (n = 3). Statistical differences were identified with unpaired t-test (*p < 0.05; ***p < 0.001; ****p < 0.0001).

The expression of hepatic markers within the PCL scaffolds was evaluated at day 20 of the differentiation. Live/dead analysis revealed that most cells were viable (Fig. 7A). The cells remained attached to the fibers and formed networks between the fibers of the scaffold, as demonstrated by F-actin staining (Fig. 7B) and SEM (Fig. 7C). Immunostainings showed that the generated cells produced albumin, mainly in 3D cellular clusters within the fibers (Fig. 7D). In addition, albumin secretion into the supernatant was detected by ELISA and showed significantly increased production of albumin (23-fold) by HLCs compared to the hiPSCs (Fig. 7E). Moreover, significantly increased expression of A1AT (428-fold), APOA2 (490-fold), and ALB (99-fold) was detected by qRT-PCR compared with the undifferentiated hiPSCs (Fig. 7F).

### Analysis of the metabolic activity of generated hiPSC-derived HLCs in PCL scaffolds

**Figure 8:**
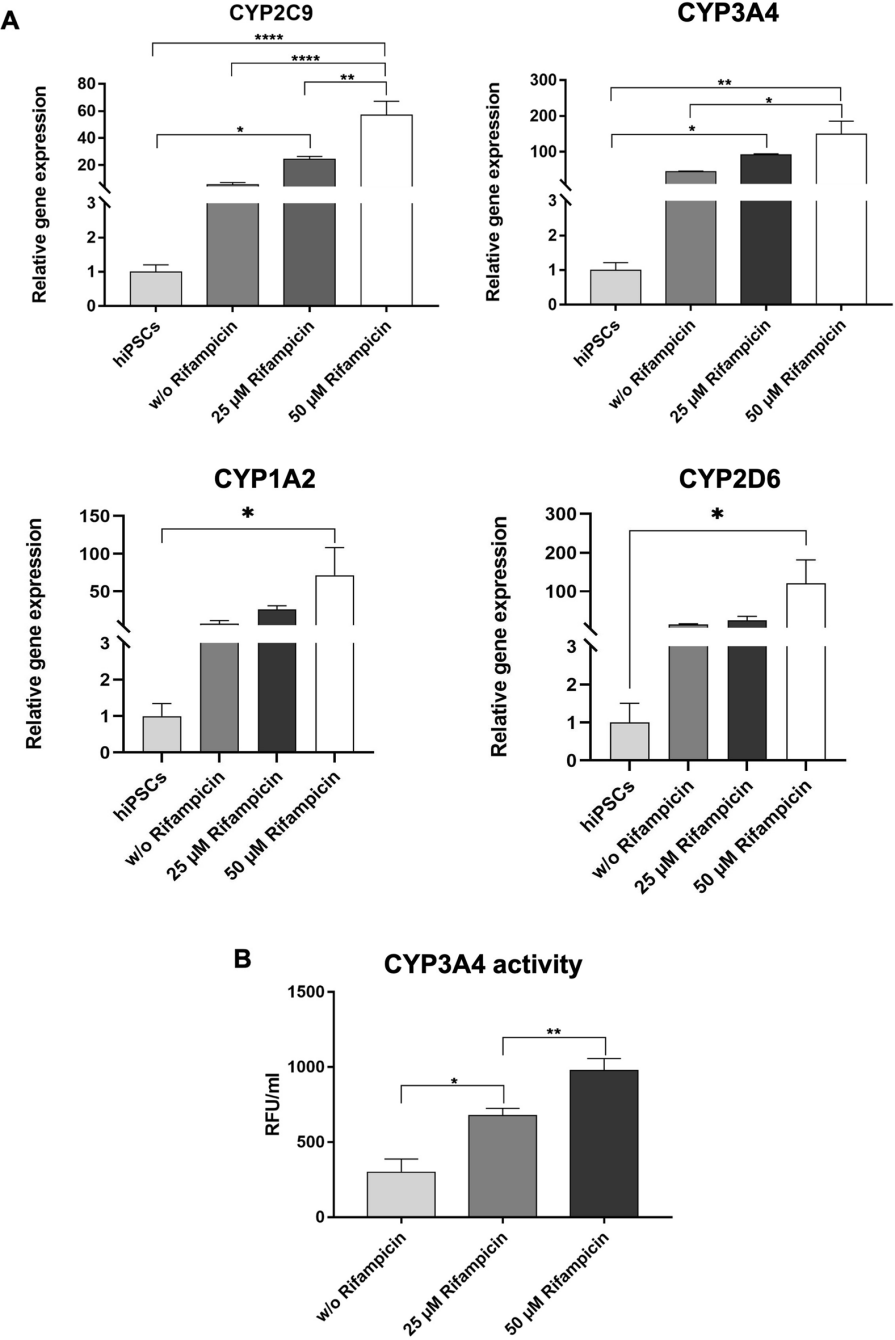
Metabolic activity of hiPSC-derived HLCs differentiated in PCL scaffolds. (**A**) Expression analysis of CYP3A4, CYP2C9, CYP1A2, and CYP2D6 transcripts performing qRT-PCR. Expression levels were examined after stimulation with 25 µM and 50 µM rifampicin for 48 h and without rifampicin treatment. (**B**) Metabolic activity of hiPSC-derived HLCs in PCL scaffolds was determined by measuring the activity of cytochrome P450 CYP3A4. All results are presented as mean + SEM (n = 3). Statistical differences were identified with one-way ANOVA (*p < 0.05; **p < 0.01; ****p < 0.0001).

To investigate the metabolic activity of generated HLCs, scaffolds with cells were treated for 48 h with 25 µM or 50 µM rifampicin. The stimulation with 50 µM rifampicin resulted in a significant increase in CYP2C9 and CYP3A4 gene expression compared with the unstimulated HLCs (Fig. 8A). Moreover, a significant increase in CYP3A4 activity was measured after the treatment of cells with 25 µM and 50 µM rifampicin (Fig. 8B).

## Discussion

Liver diseases are responsible for approximately 2 million deaths per year worldwide, of which 1 million are due to complications of liver cirrhosis and 1 million are due to viral hepatitis and hepatocellular carcinoma^28^. Thus, liver tissue engineering plays an important role in the development of novel strategies for liver regeneration and drug treatment as well as in the modeling of diseases. In this study, two key complementary components of tissue engineering were combined. First, biocompatible 3D melt-electrospun PCL scaffolds were fabricated, and second, autologous HLCs were generated from footprint-free hiPSCs to colonize these scaffolds.

The generation of patient-specific hiPSCs and the subsequent differentiation into HLCs could enable personalized treatment and liver tissue engineering by providing an unlimited source of autologous hepatocytes. A widely used strategy to obtain hiPSCs that can be further differentiated into the desired cell type, such as hepatocytes, is to reprogram skin fibroblasts using viral vectors^29,30^. However, the use of fibroblasts from skin biopsies is an invasive procedure associated with pain for the patient^23^. Additionally, genome-integrating viral vectors carry the risk of random insertion into the genome, which can potentially lead to mutations or the development of tumors^31^. Thus, meanwhile, several non-integrating reprogramming strategies have been established^32–34^. Especially, synthetic mRNA-based strategies are becoming more and more relevant^35^. The exogenous transfer of mRNAs into somatic cells results in transient expression of desired proteins. This technique can be applied for the expression of reprogramming factors for the generation of footprint-free hiPSCs^33^. Due to the transient presence of the mRNAs, a daily transfection of the cells during the reprogramming process is inevitable. This causes stress to the cells and is time-consuming and expensive. To address these challenges, srRNAs can be used for the temporary expression of the reprogramming factors^36^ without performing daily transfection^37^.

In this study, hiPSCs were used, generated by applying an innovative srRNA-based strategy. The donor-specific hiPSCs were obtained by reprogramming of urine-derived RECs with srRNA and a differentiation method was developed to obtain autologous HLCs from these footprint-free hiPSCs. After 20 days of differentiation, cells expressing hepatic lineage markers were generated. Furthermore, these hiPSC-derived HLCs exhibited functional characteristics, such as albumin synthesis, ICG metabolism, glycogen storage, and cytochrome P450 activity. Especially, CYP enzyme activity is a major function of hepatic cells, as CYP enzymes play a central role in the metabolism of clinically relevant drugs^38^. Primary hepatocytes rapidly lose their liver-specific functions, including CYP inducibility and the production of plasma proteins like albumin, during in vitro cultivation^39^. Most cell lines, e.g. HepG2^40^, also do not exhibit the natural functional properties of hepatocytes, such as CYP activities^41^. Furthermore, cancer cell lines are to some extent immature and have altered apoptotic behavior because of their tumorigenic origin, making them potentially resistant to toxicological influences and therefore inapplicable for drug screening systems^42^.

The established differentiation strategy also includes the selective elimination of undifferentiated hiPSCs by 1 h L-alanine treatment^25^ after the completion of the endodermal differentiation stage. By using this simple, inexpensive, and safe elimination method, a purified endoderm population was obtained, which promoted further differentiation efficiency.

Studies showed that cells cultured in 3D models can improve liver-specific functions compared to two-dimensional (2D) cultures due to the in vivo-like conditions mimicked by 3D models^43^. In particular, hepatic 3D models are a valuable in vitro tool for studying organogenesis, liver morphology and metabolism, but also for drug testing or cell-based assays. Thus, the development of scaffolds providing the 3D architecture to the liver cells and mimicking the extracellular matrix composition is also critical for the improvement of liver tissue engineering strategies.

PCL in particular is considered a tissue-compatible material that is also used as a biodegradable suture material^44^. Due to its semi-crystalline and hydrophobic structure, it has a slow degradation rate (2–4 years)^45^, making it suitable for long-term implants or for ex vivo biohybrid artificial liver systems^46^. It has been reported that the degradation rate of PCL can vary depending on the processing form. For example, electrospun fibers have a shorter degradation period compared to bulk PCL as a consequence of a higher surface-to-volume ratio and reduced crystallinity caused by the electrospinning process^45^. For liver tissue engineering applications, PCL can be used to enhance mechanical properties^47^. For this study PCL scaffolds were used for an in vitro cultivation period of 14 days and no degradation has been observed. However, long-term experiments must be carried out to analyze the degradation time of the scaffolds under the described conditions.

In this study, porous 3D scaffolds were fabricated by melt-electrospun PCL. This technique results in a nonwoven scaffold made of a resorbable polymer to create a microenvironment for hepatic cells using additive manufacturing methods. Other advantages of melt electrospinning include the simple equipment, and no need for solvents. The fabricated porous scaffolds can be fully penetrated with cells, which leads to the formation of an extracellular matrix within the scaffold^48^. This was found to be beneficial for the long-distance communication of cells^49^. The results of our study demonstrated that seeded hepatoblasts were uniformly attached to the PCL fibers of the scaffolds and matured into HLCs, simulating a highly simplified liver microenvironment with the self-organization of the cells. Moreover, live/dead cell analysis revealed no negative influence of the PCL scaffolds on the cells. The cells could be provided with sufficient nutrients, even in the core of the scaffolds. The hepatoblasts successfully differentiated into HLCs and exhibited hepatocyte-specific markers and functions and uniformly covered the fiber surfaces.

It has been shown that during the formation and self-assembly of artificial tissue structures, biological processes similar to those in vivo occur and help to mimic the morphological structure of native tissue^50^. In the field of liver tissue engineering, a wide variety of approaches exists to mimic the native liver microenvironment, including decellularized liver scaffolds^7^, 3D bioprinted hydrogels^8^, fiber-based 3D scaffolds^9^, or freeze-dried scaffolds^51,52^. Especially, parameters such as porosity, material and chemical properties, or scaffold architecture influence cell functions and behavior^53^. Due to their ability to mimic the in vivo microenvironment very close to nature, decellularized liver scaffolds have been frequently used for tissue engineering strategies^54^. Unfortunately, this method includes enzymatic, chemical, or physical processes to get rid of the original liver cells, to obtain a low-immunogenic scaffold^54^. Moreover, repopulated decellularized liver scaffolds often lack an intact vascular network, and bear the risk of post-transplant hemorrhage and thrombosis. But also general aspects like species^55^ or the length of scaffold preservation or storage^56^ complicate the application of decellularized scaffolds. Therefore, the generation and application of artificial scaffolds can help to overcome these challenges.

Despite the exceptional self-organizing behavior of hepatocytes in 3D environments, it remains a challenge to reproduce the complexity of the in vivo hepatic architectures, such as microvasculature, or hepatic lobules within microtissues^57^. Moreover, 3D liver tissue models including multiple cell types can further increase hepatic functions, due to their physiological interaction. However, it remains difficult to select the appropriate media and cell ratio for the co-cultivation of liver-specific cell types within one model^58^. These challenges as well as the stability and viability of the obtained hepatocytes in the scaffolds will be studied in detail over an extended time in a follow-up study.

## Conclusion

In summary, 3D biohybrid constructs were created by combining HLCs derived from patient-specific footprint-free hiPSCs and 3D melt-electrospun PCL scaffolds. The established hepatic differentiation procedure allowed the efficient and reproducible generation of HLCs from footprint-free hiPSCs in vitro. In addition, biocompatible 3D scaffolds were fabricated by melt-electrospinning of PCL, which could be uniformly populated with the generated hiPSC-derived hepatoblasts from the established in vitro differentiation procedure. Subsequent differentiation resulted in HLCs that expressed hepatocyte-specific markers and exhibited characteristic functions. In the future, these biohybrid 3D constructs could be used for ex vivo liver tissue engineering applications, disease modeling, drug testing and development. They may also represent a step towards the development of an extracorporeal hybrid bioartificial liver for the treatment of liver failure. Moreover, patient-specific footprint-free hiPSC-derived HLCs represent a promising cell source for the regeneration of the liver.

### Supplementary Information

Supplementary Figures.

## Data Availability

The datasets generated during and/or analyzed during the current study are available from the corresponding author on reasonable request.
