# Supplementary material for: Combination of melt-electrospun poly-ε-caprolactone scaffolds and hepatocyte-like cells from footprint-free hiPSCs to create 3D biohybrid constructs for liver tissue engineering
Source: Sci Rep. 2023 Dec 13;13:22174. doi: 10.1038/s41598-023-49117-x (PMC10719291; doi:10.1038/s41598-023-49117-x)
Supplement: Supplementary file 1 — Supplementary Figures. [file 41598_2023_49117_MOESM1_ESM.pdf]

Combination of melt-electrospun poly-ε-caprolactone scaffolds and hepatocyte-like cells from footprint-free hiPSCs to create 3D biohybrid constructs for liver tissue engineering

Josefin Weber[1], Carsten Linti[2], Christiane Lörch[1], Marbod Weber[1], Madelene Andt[2], Christian Schlensak[1], Hans Peter Wendel[1], Michael Doser[2], Meltem Avci-Adali[1*]

[1] University Hospital Tuebingen, Department of Thoracic and Cardiovascular Surgery, Calwerstraße 7/1, 72076 Tuebingen, Germany

[2] Biomedical Engineering, German Institutes of Textile and Fiber Research Denkendorf DITF, Körschtalstraße 26, 73770 Denkendorf, Germany

Supplementary Information 1:

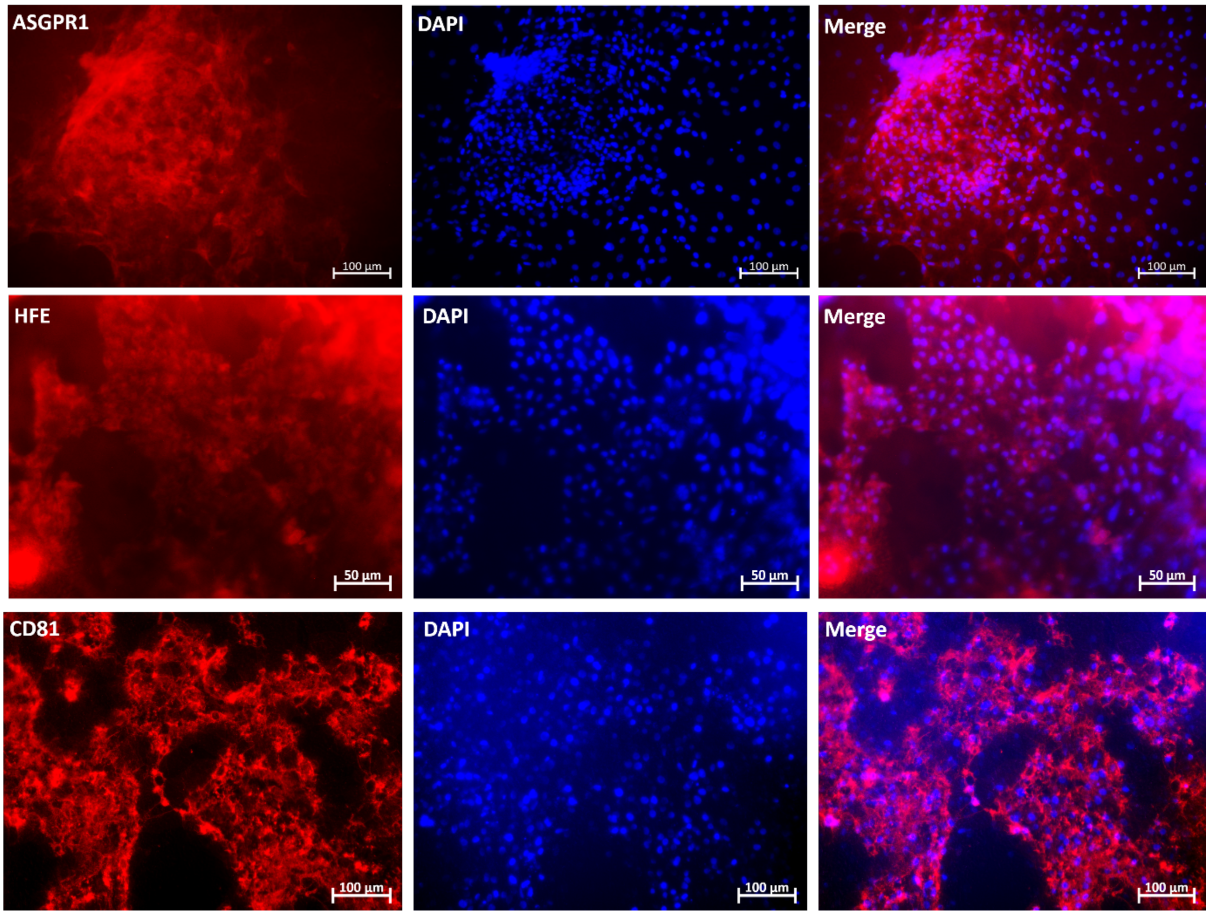

Suplementary Figure 1: Hepatic maturation and subsequent characterization of the cells. Representative immunofluorescence microscopy images of hepatocytes, stained with ASGPR1, HFE and CD81-specific antibodies. Scale bars represent 100 µm.

Supplementary Information 2:

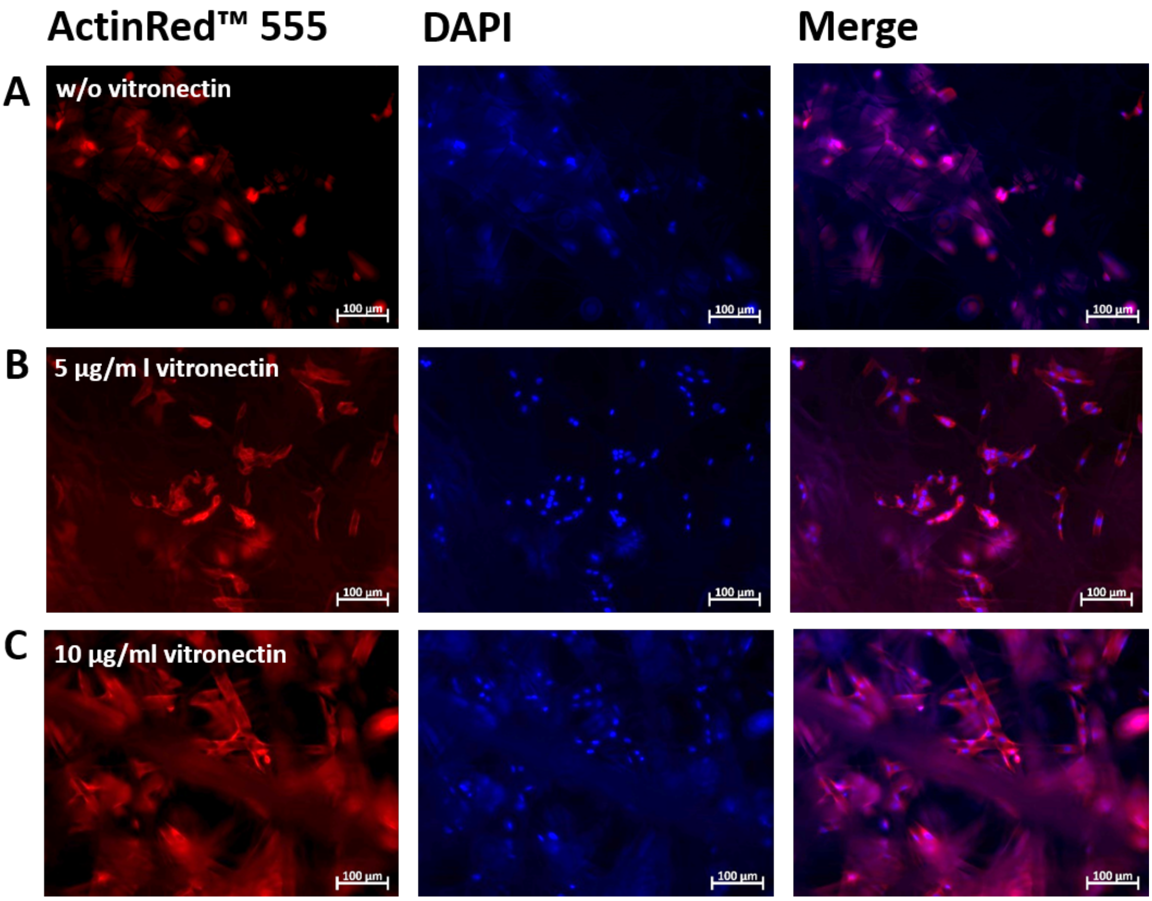

Suplementary Figure 2: Coating of PCL scaffolds with different concentrations of vitronectin. Representative immunofluorescence images of 5×10^5 HepG2 cells seeded on (A) uncoated, (B) 5 µg/ml, and (C) 10 µg/ml vitronectin-coated PCL scaffolds and stained with ActinRed™ 555. Scale bars represent 100 µm.

Supplementary Information 3:

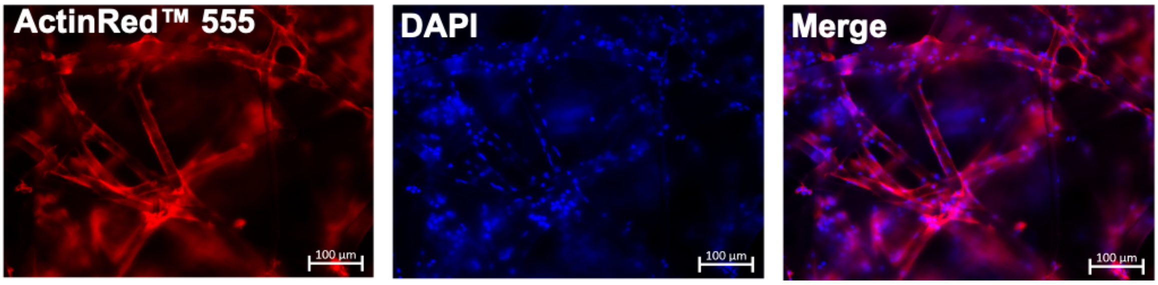

Suplementary Figure 3: Coating of PCL scaffolds with vitronectin and seeding with hepatoblasts. Representative immunofluorescence images of 5 x 10^5 hepatoblasts seeded on 10 µg/ml vitronectin-coated PCL scaffolds and stained with ActinRed™ 555. Scale bars represent 100 µm.
